# Optimal management strategies for promoting gestational extension in dichorionic diamniotic twin pregnancies

**DOI:** 10.3389/fphys.2024.1489780

**Published:** 2024-11-26

**Authors:** Caixia Chen, Changyou Fan, Bufei Wang, Ping Zhu

**Affiliations:** ^1^ Department of Obstetrics and Gynaecology, Jinan Maternity and Child Care Hospital Affiliated to Shandong First Medical University, Jinan, China; ^2^ Department of Obstetrics and Gynaecology, The First Affiliated Hospital of Shandong First Medical University, Jinan, China

**Keywords:** twin pregnancy, premature rupture of membranes, delayed interval delivery, fetal reduction, cervical cerclage

## Abstract

**Background:**

Preterm birth is a significant concern in multiple pregnancies, warranting effective strategies to improve outcomes. Delaying delivery of the second fetus is crucial for reducing perinatal mortality rates.

**Case Presentation:**

In a dichorionic diamniotic twin pregnancy, one fetus experienced premature rupture of membranes (PROM) at 16+6 weeks gestation. Proactive fetal reduction through potassium chloride injection and emergency cervical cerclage at 19+1 week successfully extended the pregnancy to 39+5 weeks, resulting in a notable 160-day prolongation. Postoperative management encompassed comprehensive tocolytic therapy.

**Conclusion:**

The combined approach of proactive fetal reduction and emergency cervical cerclage proved successful in managing PROM in dichorionic diamniotic twin pregnancies. This innovative strategy offers a promising clinical solution for optimizing outcomes and prolonging gestation in high-risk multiple pregnancies, underscoring the importance of tailored interventions in complex obstetric scenarios.

## Introduction

In recent years, with the widespread use of assisted reproductive technology (ART), the incidence of multiple pregnancies has significantly increased worldwide. According to the latest data, multiple pregnancies now account for over 3.2% of all pregnancies, with an even higher proportion among those using ART (Gestations and Twin, 2021; Sunderam et al., 2022). Twin pregnancies are often associated with a higher risk of pregnancy complications, particularly preterm birth and premature rupture of membranes (PROM), both of which are closely linked to increased neonatal morbidity and mortality (Weitzner et al., 2023; Khalil and Prasad, 2022; Cheung et al., 2020). As a result, effective management of multiple pregnancies to reduce maternal and neonatal complications has become a key focus of clinical research. The most effective way to prevent preterm birth is to promptly identify high-risk individuals and implement decisive interventions, such as bed rest or restricted physical activity, the use of tocolytics, uterine pessaries, and cervical cerclage (Roman et al., 2022).

Delayed interval delivery (DID), a method for prolonging pregnancy, was first introduced in the 1960s (ABRAMS, 1957) and has gained significant attention in recent years. DID refers to the delivery of the first fetus in a multiple pregnancy while delaying the delivery of the remaining fetus or fetuses (Cheung et al., 2020). Recent studies have shown that DID can significantly extend the gestational period and improve perinatal outcomes in twin pregnancies (Cheung et al., 2020; ABRAMS, 1957). It effectively reduces the risk of preterm birth for the second fetus in multiple pregnancies and increases survival rates. A meta-analysis by Cheung et al. (2020) found that DID can raise the fetal survival rate to 87.5% and decrease the incidence of adverse pregnancy outcomes (Kolben et al., 2019). These findings suggest that DID can be an effective intervention under certain conditions. However, due to the complexity and variability of clinical cases, the implementation of DID still lacks standardized protocols and guidelines (de Frias et al., 2020). A systematic review showed that the use of DID strategies in multiple pregnancies can extend the gestational period by an average of 30–42 days, significantly reducing perinatal complications (Cheung et al., 2020). DID markedly decreases the incidence of maternal and neonatal complications, significantly improving perinatal outcomes (Kolben et al., 2019). The literature review indicates that implementing DID positively correlates with favorable neonatal outcomes. The systematic review data reveal that when DID is applied between 24 and 28 weeks of gestation, the survival rate of the second fetus increases from 50% to 80%, with a significant reduction in complication rates. Furthermore, the latest meta-analysis highlights that the effectiveness of DID is positively correlated with the duration of gestational extension, with survival rates increasing by 5% for every 10 additional days of pregnancy (Feys and Jacquemyn, 2016). These findings further underscore the potential value of DID in improving perinatal outcomes. However, there are no standardized treatment protocols or guidelines for this method, and no randomized controlled trials have been reported (Cheung et al., 2020). Although there have been numerous studies on DID, a meta-analysis by Cheung et al. (2020) demonstrated that implementing DID in multiple pregnancies between 13 + 0 and 31+6 weeks of gestation can increase survival rates to 87.5% and significantly reduce the incidence of adverse pregnancy outcomes (Cheung et al., 2020). Additionally, a systematic review of 50 DID cases from 1980 to 2020 found that DID can extend gestation by an average of 30–42 days and reduce preterm-related complications by 28% (Kolben et al., 2019). However, significant gaps remain in understanding the potential risks of DID, such as intrauterine infection and neonatal sepsis, as well as the benefits of DID concerning mortality and short- and long-term morbidity.

In this case report, we documented a rare instance of dichorionic diamniotic twin pregnancy where one fetus experienced PROM. By employing a combined strategy of fetal reduction and emergency cervical cerclage, the gestation of the remaining fetus was successfully extended by 160 days, resulting in a healthy birth. This case not only provides crucial clinical evidence of the potential value of DID but also offers new perspectives and insights for the development of future management strategies for multiple pregnancies (Cui et al., 2024). Furthermore, supported by the latest literature from the past 3 years, this study provides essential theoretical foundations and data for clinical practice, underscoring the necessity and effectiveness of comprehensive treatment strategies in managing complex multiple pregnancies.

## Cases

**FIGURE 1:**
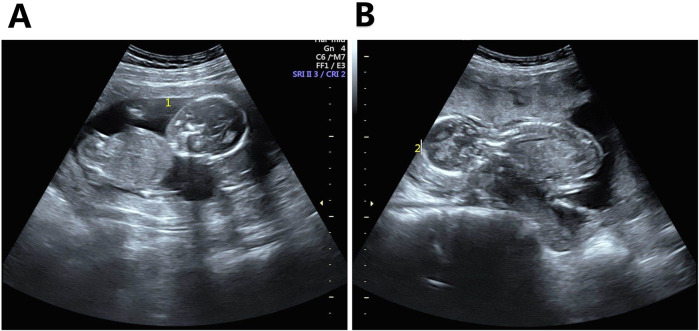
Ultrasound images of the two fetuses before fetal reduction. Note: **(A)** Amniotic cavity of Fetus 1 showing almost no amniotic fluid; **(B)** Normal amniotic fluid volume in Fetus 2.

The patient, a 33-year-old woman, was admitted to our hospital on 5 May 2024, with complaints of irregular lower abdominal pain lasting for 2 hours at 39+5 weeks of gestation. She has a history of a complete septate uterus and underwent hysteroscopic septum resection at an external hospital in 2022, with a good recovery. It is her first pregnancy (G1P0). Her last menstrual period was on 2 August 2023, with an expected delivery date of 9 May 2024. On 17 August 2023, she underwent IVF-ET at an external hospital due to primary infertility, where two fresh embryos were transferred, followed by progesterone therapy for luteal support. An ultrasound at 8+ weeks of gestation confirmed an intrauterine twin pregnancy, dichorionic diamniotic, consistent with an 8-week gestation size. The patient had no other pregnancy-related complications throughout the entire gestation and gained 15.7 kg, with a pre-pregnancy BMI of 19.36 kg/m^2^. At 16+6 weeks, one fetus experienced PROM with clear amniotic fluid. Ultrasound revealed oligohydramnios, with minimal amniotic fluid visible, while the other fetus had a maximum amniotic fluid depth of 4.9 cm and was growing appropriately for the gestational age (Figure 1). The patient did not experience significant abdominal pain or other discomforts and was thus treated with tocolytics and antibiotics for infection prevention as part of comprehensive pregnancy maintenance.

**FIGURE 2:**
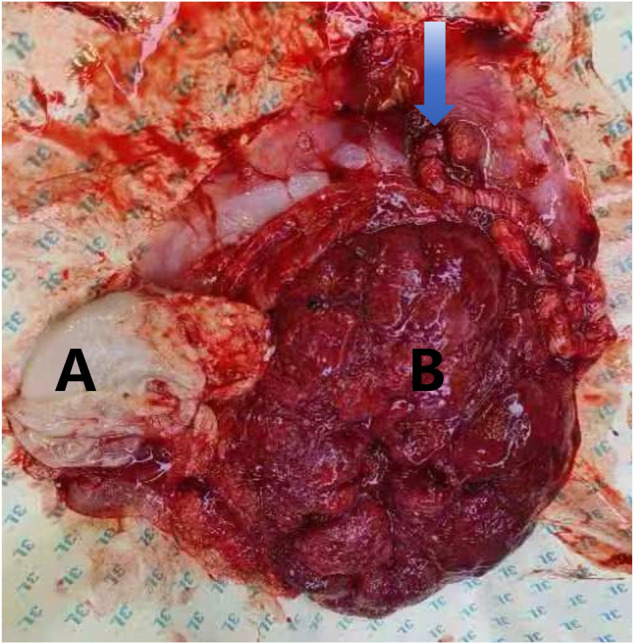
Placental images following fetal reduction in dichorionic diamniotic twin pregnancy. Note: **(A)** The arrow indicates the umbilical cord insertion point and excessive cord twisting. The uterine cavity contains a deceased fetus; **(B)** approximately 8 cm in length. The surviving fetus has a normal placenta.

**FIGURE 3:**
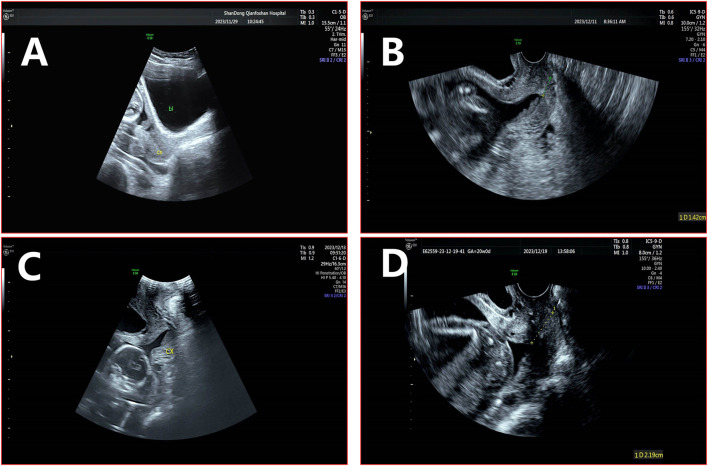
Cervical canal before and after cerclage. Note: **(A)** The internal cavity of the cervical canal remains closed during preterm PROM. **(B)** Before cervical cerclage, the internal os is V-shaped with an opening of approximately 2.7 × 1.4 cm, with a remaining cervical canal length of 1.4 cm. **(C)** The cervical canal is fully open, with an internal os width of approximately 0.5 cm. **(D)** After cervical cerclage, the internal os is V-shaped with a smaller opening of approximately 0.9 × 0.7 cm, and the remaining cervical canal length is approximately 2.2 cm.

Before performing the fetal reduction, a comprehensive preoperative evaluation was conducted, including a detailed analysis of the patient’s medical history, imaging studies to assess fetal growth and amniotic fluid volume, and necessary laboratory tests to ensure the patient’s suitability for surgery. Preoperative blood tests showed that the patient’s white blood cell count and C-reactive protein (CRP) levels were within normal ranges, indicating no signs of infection. At 17+6 weeks of gestation, the patient’s condition was stable, with no signs of infection or significant uterine contractions. After a thorough discussion with the patient and her family, who strongly desired to continue the pregnancy, informed consent was obtained for the fetal reduction. The procedure for intra-abdominal fetal heart injection of potassium chloride for reduction is performed under local anesthesia, with real-time ultrasound monitoring. A puncture needle is inserted into the target fetus’s heart or near the heart. Once the needle reaches the correct position and aspirates, confirming it is within the heart, potassium chloride solution is slowly injected. The dosage used ranges from 1.5 to 7.5 mL. After the injection, the fetal heartbeat is monitored via ultrasound until fetal heart activity ceases and fetal movement disappears, ensuring successful reduction. Throughout the procedure, fetal heart rate and maternal vital signs were closely monitored to ensure safety and effectiveness. Postoperatively, the patient’s vital signs and vaginal bleeding were carefully observed, antibiotics were administered to prevent infection, and psychological support was provided to help the patient cope with the emotional challenges of the procedure. The reduction was confirmed successfully during surgery (Figure 2). Two days after the procedure, an ultrasound confirmed normal umbilical blood flow and amniotic fluid volume in the remaining fetus. Cervical length was dynamically monitored after the reduction, and at 19+1 week, a transvaginal ultrasound revealed that the cervical canal was fully open, with an internal os width of approximately 0.55 cm and a length of 2.9 cm (Figures 3A, B). Vaginal speculum examination showed that the cervical canal was nearly effaced, with the cervix dilated to 2 cm, exposing the amniotic sac, though the membranes remained intact, and the pH test strip showed no color change. A definitive diagnosis of cervical incompetence was made, leading to the performance of a McDonald’s cervical cerclage via the vaginal route under epidural anesthesia.

Before performing the emergency cervical cerclage, a thorough assessment of the cervical condition was conducted, and the risk of intrauterine infection was ruled out through transvaginal ultrasound. The cervical cerclage was performed using the McDonald technique. During the procedure, the cervix was exposed with the assistance of a speculum. Once the cervix was revealed, cervical forceps were used to grasp the cervix and apply downward traction. A 7 or 10-gauge double-stranded suture or nylon thread was used; the needle was inserted at the bladder attachment just below the cervix at the 11 o’clock position, passed through the cervical mucosa and muscular layer, and then exited at the 10 o’clock position. The cervical forceps were used to pull the cervix upward, and the suture continued to create a purse-string suture at the 7-8 o’clock, 4-5 o’clock, and 1-2 o’clock positions. The sutures were tightened and tied at the anterior fornix, ensuring that the amniotic sac remained intact and was not perforated.

Postoperatively, the patient was required to strictly rest in bed, monitoring for uterine contractions and changes in vaginal discharge while being vigilant for signs of preterm labor. Long-term management and regular prenatal check-ups were planned to continuously observe cervical changes and fetal development, with timely adjustments to the preservation treatment protocol. During the procedure, it was noted that the cervical canal was flattened, and the external os was dilated to 2 cm, with the amniotic sac protruding. A single-use cervical balloon dilator was used to reduce the amniotic sac, and the surgery proceeded smoothly, followed by proactive preservation treatment.

Postoperative care included strict bed rest and maintaining a position with the head lower than the hips to reduce cervical pressure. Vital signs, including temperature, pulse, respiration, blood pressure, and fetal heart rate, were monitored every 4 h for the first 2 days and then every 8 h thereafter to ensure maternal and fetal wellbeing.

Regarding medication management, the patient was given oral progesterone soft capsules, 400 mg twice daily, to support luteal function and suppress uterine contractions. Broad-spectrum antibiotics, preferably cephalosporins, were administered to prevent infection. Magnesium sulfate was used to control uterine contractions for the first 5 days, with a dosage of 5 g every 6 hours administered via intravenous infusion, and serum magnesium levels were monitored to prevent overdose. In terms of dietary care, the patient was advised to consume a high-protein, high-fiber, low-fat diet to enhance strength and promote postoperative recovery. Increased fluid intake was encouraged to prevent urinary tract infections and constipation. Postoperatively, the patient received routine antibiotic treatment to prevent infection. Given the risk of infection following cervical cerclage, her temperature, white blood cell count, and CRP levels were closely monitored within the first 48 h after surgery, with results indicating no signs of infection. Uterine contractions were well-controlled postoperatively, with no significant increase in intensity or frequency, and progesterone was continued to stabilize the uterine environment.

However, on the fifth postoperative day, the patient experienced mild abdominal pain. A follow-up transvaginal ultrasound revealed that the internal os was slightly V-shaped, with an opening of approximately 0.9 × 0.7 cm and a remaining cervical canal length of 2.2 cm (Figures 3C, D). It was assessed as physiological uterine contractions, which did not progress to sustained contractions. Observation and symptomatic treatment were continued. Blood tests showed a slight increase in white blood cell count, but it remained within the normal range, while CRP levels were mildly elevated, indicating a slight inflammatory response. By the seventh postoperative day, follow-up tests showed that the white blood cell count and CRP levels had returned to normal, indicating a good postoperative recovery with no signs of infection. The patient was then discharged.

After discharge, the patient attended regular outpatient follow-ups to monitor cervical length and fetal growth. Transvaginal ultrasound indicated progressive shortening of the cervix, while the retained fetus continued to grow normally, and the amniotic fluid index remained within normal limits. Throughout the follow-up period, there were no significant signs of infection, and cultures of vaginal secretions showed no bacterial growth, indicating no infection. The patient did not experience any severe complications, cervical length remained stable, and uterine contractions were well-controlled. Vaginal progesterone soft capsules and oral Anbao were continued for pregnancy maintenance.

At 38 weeks of gestation, the patient returned to the hospital to have the cervical cerclage removed. At 39+5 weeks, she was admitted to the hospital due to irregular lower abdominal pain, accompanied by bloody show but without vaginal bleeding or rupture of membranes. She reported no dizziness, visual disturbances, chest tightness, or shortness of breath. The patient was in good spirits, had a good appetite, had good sleep quality, and had normal bowel and bladder function. Upon admission, her vital signs were as follows: temperature 36.2°C, pulse 74 beats per minute, respiration 19 breaths per minute, and blood pressure 125/75 mmHg. Her pre-pregnancy weight was 54 kg, with a BMI of 19.36 kg/m^2^, and she had gained 15.7 kg during pregnancy. Cardiopulmonary examination revealed no abnormalities, the abdomen was soft without tenderness, the uterine fundal height was 39 cm, and the abdominal circumference was 96 cm. The fetal heart rate was 150 beats per minute in the left occiput anterior (LOA) position, with the engagement of the fetal head. There were irregular contractions, occurring every 5–10 min, lasting 20–30 s with moderate intensity. Non-stress test (NST) results were reactive. Vaginal examination revealed that the cervical canal had effaced by 50%, with a medium consistency, posterior position, and 1 cm dilation of the cervical os, S-3. The fetal membranes were intact, and no amniotic fluid was visible.

Admission Diagnosis: (1) 39+5 weeks gestation, in labor, G1P0L0A0, LOA; (2) post-fetal reduction of one twin in a dichorionic diamniotic twin pregnancy; (3) post-cervical cerclage removal; (4) post-IVF-ET. After admission, uterine contractions gradually became regular, and pelvic measurements showed no significant abnormalities. A trial of labor was conducted under close monitoring. However, due to suspected fetal distress, a lower segment cesarean section was performed under combined spinal-epidural anesthesia on 10 May 2024, at 06:20. During the surgery, clear amniotic fluid was observed and suctioned. At 06:23, a full-term newborn was delivered in the LOA position, weighing 2950 g, with Apgar scores of 10 at 1, 5, and 10 min. The umbilical cord was excessively twisted, about the thickness of a small finger and approximately 55 cm long, located beside the fetal head.

Inside the uterine cavity was a lithopedion measuring approximately 8 cm in length. The placenta and membranes were delivered intact, and a portion of the membranes near the internal cervical os was sent for pathological examination. Oxytocin (10 units) was injected into the uterine, producing good uterine contraction. Intraoperative exploration revealed adhesions between the posterior uterine wall and portions of the intestines, with membranous adhesions covering the surfaces of both ovaries and fallopian tubes. Adhesions at the fimbrial ends of the fallopian tubes were dissected and found to be adherent to the ovarian surfaces. The surgery proceeded smoothly, with an estimated blood loss of approximately 400 mL.

Postoperatively, the patient was treated with fluids, antibiotics for infection prevention, and uterotonic agents. The newborn’s physical examination showed no abnormalities, and both mother and child were discharged as scheduled. Prenatal, postnatal, and membrane-related microbiological tests were all negative, and the pathological examination of the membranes revealed grade III acute chorioamnionitis.

After the patient was discharged, regular postoperative follow-ups were conducted to monitor her *postpartum* physical condition, uterine recovery, and maternal and neonatal health. Uterine evaluations at 1 week and 1 month *postpartum* revealed that the uterus gradually returned to its normal size, with a stable cervical structure. Regarding maternal and neonatal health, the patient showed no significant complications during *postpartum* checkups. The newborn received Apgar scores of 10 at 1, 5, and 10 min after birth, indicating excellent vital signs. At the 6-week *postpartum* follow-up, the mother reported good physical recovery, a stable mental state, and no signs of anxiety or depression. The newborn’s growth and development were average, with regular follow-ups showing that weight and height increases were consistent with age standards, and no infections or other health issues were observed. Follow-up survey results showed that the mother and baby were healthy 5 months *postpartum*, with no significant complications observed. Throughout the entire follow-up period, the patient did not experience any complications that required rehospitalization or emergency care, demonstrating the effectiveness of the surgical management and postoperative care.

## Discussion

### The increase in multiple pregnancies and ART

With the widespread use of ART, such as *in vitro* fertilization and ovulation-inducing drugs, the incidence of multiple pregnancies has significantly increased worldwide (Gestations and Twin, 2021). Recent data from a 2018 study in the United States indicate that 21.4% of infants conceived through ART were multiples, a rate considerably higher than the 3.3% observed in the general population. Specifically, among 74,926 ART pregnancies, 20.7% (15,532 cases) resulted in twins, and 0.6% (469 cases) in triplets or higher-order multiples (Sunderam et al., 2022). A retrospective study involving 12,000 twin pregnancies found that using cervical cerclage and tocolytic agents improved the success rate of DID to 65% and increased the twin survival rate to 75% (Sunderam et al., 2022). This study further highlighted that timely cervical cerclage in the context of DID significantly reduces maternal infection rates (Bell et al., 2022). Multiple pregnancies pose significant challenges for both women and infants, as they increase the risk of pregnancy complications, with preterm birth being one of the most critical, leading to higher neonatal morbidity and mortality (Khalil and Prasad, 2022; Roman et al., 2022; Weitzner et al., 2023; Khalil and Liu, 2021; Bell et al., 2022). The case presented in this study demonstrates that the combination of fetal reduction and emergency cervical cerclage can significantly prolong gestation and reduce the risk of preterm birth. Zhang et al. reported that implementing such a comprehensive strategy significantly extended the delivery interval and reduced infection rates (Zhang et al., 2003).

### Preterm birth and its associated complications

Preterm birth is associated with a range of complications, including respiratory distress syndrome (RDS), sepsis, necrotizing enterocolitis (NEC), and cerebral palsy (McDonnell and Martin, 2022). The risk of perinatal morbidity and mortality is inversely correlated with the gestational age of preterm infants (Cheung et al., 2020). Complications related to preterm birth are currently the leading cause of death and disability among children under 5 years old, causing immeasurable trauma to parents and families and leading to significantly increased healthcare costs (Ohuma et al., 2023). Addressing the global burden of preterm birth is crucial for achieving the United Nations' 2030 Sustainable Development Goal 3-2, which aims to end preventable deaths of newborns and children under five, with countries striving to reduce neonatal mortality to at least 12 per 1,000 live births (Yang et al., 2022). Timely identification of high-risk populations and the implementation of decisive and effective interventions may be the most effective strategies for preventing preterm birth. Common preventive measures in twin pregnancies include bed rest or activity restriction, the use of tocolytic agents, the placement of a pessary, and cervical cerclage (Roman et al., 2022). However, despite these interventions, the overall rate of preterm birth and obstetric outcomes have remained relatively unchanged, indicating limited effectiveness and ongoing controversy regarding these approaches (Bell et al., 2022; ACOG, 2014). There is an urgent need for large-scale, multicenter epidemiological studies to explore further effective preventive measures for preterm birth in twin pregnancies.

### The combined use of fetal reduction and emergency cervical cerclage

The combined application of fetal reduction and emergency cervical cerclage has been proven to significantly prolong pregnancy duration and reduce the risk of preterm birth. In the management of multiple pregnancies, fetal reduction is a common measure to reduce adverse pregnancy outcomes. Most of the reported cases of DID are passive DID, occurring when a twin pregnancy attempts to prolong the intrauterine duration of the remaining fetus after spontaneous delivery of the first fetus.

In this case, after PROM in one fetus, effective uterine contractions were not induced, and both fetuses remained *in utero*. To avoid complications such as chorioamnionitis, we proactively selected fetal reduction for the fetus with PROM. Following the reduction, due to the interruption of maternal-fetal circulation, amniotic fluid was no longer leaking. Clinically, fetal reduction is a relatively common practice to reduce the occurrence of adverse pregnancy outcomes in multiple pregnancies. Previous studies have shown that fetal reduction can significantly reduce the risk of spontaneous preterm birth and other maternal-fetal complications in multiple pregnancies (Bardin et al., 2022; van de Mheen et al., 2015; Hessami et al., 2022). Reducing a twin pregnancy to a singleton can lower the risk of preterm birth before 37 weeks, though it does not reduce the risk of perinatal complications (Greenberg et al., 2020). A retrospective cohort study from Denmark found that fetal reduction in dichorionic twin pregnancies due to fetal abnormalities or severe maternal complications was proven to be safe and effective. Compared with non-reduced twin pregnancies, the overall incidence of pregnancy complications decreased by 50% in cases where fetal reduction was performed, with a 4.1% risk of adverse pregnancy outcomes, and the overall preterm birth rate was significantly lower than in non-reduced twin pregnancies. The study also recommended that fetal reduction be performed before 14 weeks, as a significant negative correlation exists between the gestational age at reduction and the gestational age at delivery (Kristensen et al., 2023). This is consistent with Vieira LA et al. (Vieira et al., 2019), who demonstrated that patients who reduced from twin to singleton pregnancies had higher gestational ages at delivery and lower rates of preterm birth and pregnancy complications without an increased risk of pregnancy loss.

The routine use of cervical cerclage in DID patients is controversial. The key concern is the safety of cervical cerclage: the procedure itself may induce uterine contractions, cause PROM, or lead to perioperative infection, all of which are unfavorable for prolonging the interval between deliveries (Arabin and van Eyck, 2009; Benito Vielba et al., 2019). Initially, we did not immediately perform cervical cerclage after fetal reduction due to concerns that the procedure might increase the risk of intrauterine infection and PROM. However, on the ninth day after reduction, examination revealed cervical dilation and protrusion of the amniotic sac. To prevent miscarriage, we performed emergency cervical cerclage after ruling out intrauterine infection.

In recent years, many studies have described the successful application of cervical cerclage in DID cases. Zhang et al. (2003) found that emergency cervical cerclage significantly prolonged the delivery interval in cases of delayed delivery, and after controlling for factors such as gestational age, antibiotics, and tocolytics, emergency cerclage did not increase the risk of intrauterine infection. Cui et al. (2024) conducted a systematic review of the application of cervical cerclage in DID pregnancies, identifying 22 articles that met inclusion criteria. The results showed that patients who received cervical cerclage had a longer delivery interval compared to those who did not and a lower incidence of chorioamnionitis and maternal complications. It is hypothesized that cervical cerclage helps to close the dilated cervix, reducing the exposure of the amniotic sac to bacteria and the acidic vaginal environment while also increasing cervical stability, thus reducing the risks of PROM and chorioamnionitis. In this case, changes in white blood cell count and C-reactive protein levels observed in pre-and post-operative blood tests further supported the effectiveness of emergency cervical cerclage in controlling infection and promoting postoperative recovery.

### DID strategy and its application

In cases of twin or multiple pregnancies, following the natural delivery of one fetus, uterine contractions often persist, leading to miscarriage or preterm delivery of the remaining fetuses, which can result in pregnancy failure. DID refers to the delivery of the first fetus in a multiple pregnancy while delaying the delivery of the remaining fetuses (Cheung et al., 2020). This technique, first reported in the 1960s as a means to extend pregnancy following the natural delivery of the first fetus, has garnered attention over the past few decades (Cheung et al., 2020). Numerous case reports, small case-control studies, cohort studies involving up to 50 cases, and systematic reviews related to DID have been published (Cheung et al., 2020; McDonnell and Martin, 2022; de Frias et al., 2020; Zhang et al., 2022; Feys and Jacquemyn, 2016). Previous case reports have shown that not performing cervical cerclage after the first delivery did not reduce the risk of severe preterm birth, and there was no significant improvement in prognosis or perinatal outcomes (Benito Vielba et al., 2019). Another multicenter study indicated that DID might extend the gestational period for the second set of twins and allow for successful delivery of the second set; however, there is a risk of maternal complications (Louchet et al., 2020). The gradual implementation of DID in multiple pregnancies has significantly reduced the incidence of maternal and neonatal complications, leading to considerable improvements in perinatal outcomes (Kolben et al., 2019). The reported average interval between deliveries in DID cases ranges from 12 to 42 days, with the longest recorded interval extending to 154 days (Cheung et al., 2020; de Frias et al., 2020). Previous studies have not adequately discussed the impact of management strategies combining DID and cervical cerclage on prognostic outcomes. This study suggests that the combination of DID and cervical cerclage may be beneficial in improving prognostic outcomes. However, there remains a lack of large-scale studies that systematically describe DID protocols, maternal morbidity and mortality, and both short- and long-term outcomes for the children. There are no standardized treatment protocols or guidelines for this approach. Furthermore, no randomized controlled trials have been conducted, and there remains a significant knowledge gap regarding the balance between the potential risks of DID, such as intrauterine infection and neonatal sepsis, and its benefits in terms of reducing mortality and short- and long-term morbidity.

### Management measures and controversies in DID

The standardized management of DID has yet to gain widespread acceptance, and the optimal treatment protocol remains under exploration. Common clinical management measures for DID include bed rest, high ligation of the umbilical cord of the delivered fetus, infection prevention, fetal lung maturation therapy, judicious use of tocolytics, magnesium sulfate for fetal neuroprotection, and emergency cervical cerclage. However, the effectiveness and safety of these interventions remain highly controversial.

High ligation of the umbilical cord of the delivered fetus using absorbable sutures under sterile conditions is recommended by many scholars, primarily to prevent ascending infections, which are the most common complications of DID and the leading cause of DID failure (Feys and Jacquemyn, 2016; Zheng et al., 2020). Studies indicate that approximately 22% of DID patients develop inflammatory conditions such as chorioamnionitis, thrombophlebitis, and endometritis, with nearly 10% progressing to *postpartum* hemorrhage and 6% experiencing placental abruption (Arabin and van Eyck, 2009). Given these risks, using antibiotics to prevent infection is particularly crucial. However, there is no consensus on the choice of antibiotics, duration of treatment, or administration routes, and no standardized regimen has been established in clinical practice. Most studies recommend selecting antibiotics based on cervical secretion culture results. However, due to the delay in obtaining lab results and the potential for false negatives, clinicians primarily rely on broad-spectrum antibiotics for prophylactic treatment (Raposo et al., 2017; Yodoshi et al., 2015).

The rational use of tocolytic drugs is a critical factor in determining the success of DID. The primary goal of these medications is to inhibit uterine contractions, thereby delaying preterm birth, allowing time for fetal lung maturation, and facilitating *in utero* transfer, ultimately reducing morbidity and mortality in preterm infants. However, the efficacy and safety of tocolytics remain uncertain. A meta-analysis of 122 trials involving 13,697 women indicated that, compared to placebo or no treatment, all tocolytic agents—whether used alone or in combination—might effectively delay preterm birth (Cheung et al., 2020). Haas et al. (2012) conducted a systematic review and network meta-analysis of 95 randomized controlled trials on tocolytic therapy, finding that prostaglandin inhibitors and calcium channel blockers had the highest potential for delaying delivery and improving neonatal and maternal outcomes. However, both the American College of Obstetricians and Gynecologists (ACOG) and the FDA have repeatedly emphasized that prolonged use of tocolytics does not significantly prevent preterm birth or improve neonatal outcomes. Typically, it is recommended that tocolytic therapy should not exceed 48–72 h, as the combined use of two or more tocolytics may increase the risk of adverse effects, and thus, combination therapy should be avoided (American College of Obstetricians and Gynecologists’ Committee on Practice Bulletins—Obstetrics, 2016). In clinical practice, tocolytics should be individualized based on the patient’s circumstances, including gestational age, medical history, severity of adverse reactions, and response to treatment.

Strict bed rest is also commonly recommended, sometimes even involving the Trendelenburg position (head down, pelvis elevated) to prevent preterm birth. However, this is often difficult to implement effectively due to low patient compliance. A Cochrane review found that for women with uncomplicated twin pregnancies, routine hospitalization or bed rest did not provide significant benefits (da Silva Lopes et al., 2017). Additionally, the ACOG practice bulletin advises against routine hospitalization and bed rest for women with uncomplicated twin pregnancies, as prolonged bed rest may increase the risks of thromboembolism and muscle atrophy (Gestations and Twin, 2021).

The ethical issues arising from fetal reduction warrant clinical attention. The implementation of fetal reduction requires careful consideration of the moral status and rights of the fetus. Some argue that the fetus, as a potential human, should be regarded as a potential subject of human rights and entitled to certain limited rights; any fetus treatment should occur within a strict ethical and legal framework (Cheong and Tay, 2014). In the ethical decision-making process, informed consent from the patient is crucial. Physicians must thoroughly explain the procedure’s necessity, risks, and potential outcomes to ensure that patients make decisions based on comprehensive information. Additionally, physicians should consider patients' values and wishes, respecting their choices (Chen et al., 2021). These controversial issues highlight the need for further research into DID management to establish more scientifically sound and consistent treatment protocols, ensuring the safety and effectiveness of care for both mother and child.

Currently, the widely accepted contraindications for DID include placental abruption, placenta previa, preeclampsia, fetal anomalies, PROM in the remaining fetus, intrauterine infection, and other conditions that necessitate the termination of pregnancy (Cristinelli et al., 2005). The primary controversies surrounding DID focus on the gestational age at which it is performed and the chronicity of the pregnancy. Gestational age at delivery is a critical predictor of outcomes for preterm infants. Deliveries before 24 weeks of gestation are associated with poor prognoses for the fetus. However, extending gestation and increasing fetal weight can significantly improve outcomes, leading more researchers to explore DID with promising results. Despite this, there is no consensus on the optimal gestational age for implementing DID. Porreco et al. (1998) suggest that preterm infants born after 28 weeks of gestation have a higher survival rate with aggressive neonatal care, but DID beyond 28 weeks may increase the risk of severe maternal and fetal complications, such as placental abruption and chorioamnionitis, potentially leading to adverse pregnancy outcomes. Therefore, they do not recommend DID after 28 weeks of gestation. Conversely, a prospective study by Arabin and van Eyck (2009) indicates that performing DID in twin pregnancies with gestational ages between 20 and 29 weeks can significantly improve outcomes for the retained fetus. In a 2020 meta-analysis by Cheung et al. (2020), it was noted that when the first fetus is delivered between 13 + 0 and 31+6 weeks of gestation, DID is an effective strategy for improving the survival rate of the remaining fetus. However, the mortality rate of the remaining fetus must also be considered. Kolben et al. (2019) reported in a study of 14 DID cases that the mortality rate for the first fetus was 53.3%, while the mortality rate for the second fetus was 17.6%. Although DID positively impacts the extension of gestation and neonatal survival rates, the short-term and long-term outcomes for neonates remain unclear. A case-control study involving 17 DID cases found that the average delayed interval was 36 days, with a first-fetus mortality rate of 76% and a second-fetus mortality rate of 5.8%. The short-term and long-term morbidity rates of the second fetus did not differ significantly from those of age-matched controls (Zhang et al., 2022). Other studies also indicate that the short- and long-term neurodevelopmental outcomes of DID fetuses are comparable to those of age-matched children (Raposo et al., 2017; Kaneko et al., 2012). In summary, although there is no standardized guideline for DID, most studies support considering DID when the gestational age is less than 28 weeks and no contraindications are present. Before implementing DID, a thorough and comprehensive evaluation of maternal and fetal conditions and the level of care available in the NICU should be conducted to achieve better perinatal outcomes.

## Recommendations for future research and clinical practice

**FIGURE 4:**
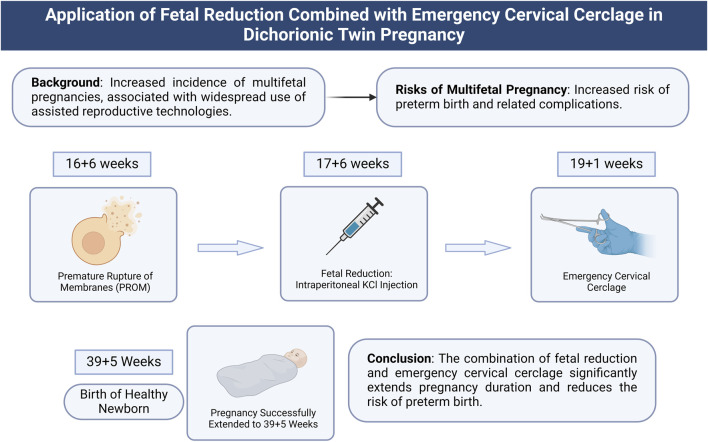
Application of combined fetal reduction and emergency cervical cerclage in dichorionic diamniotic twin pregnancy. Note: This illustration depicts the treatment process and outcomes of combining fetal reduction and emergency cervical cerclage in a dichorionic diamniotic twin pregnancy. The background information highlights the increased incidence of multiple pregnancies due to the widespread use of ART, which elevates the risk of preterm birth. During the study, PROM occurred at 16+6 weeks, followed by fetal reduction via intrafetal potassium chloride injection at 17+6 weeks to remove the fetus with ruptured membranes. Emergency cervical cerclage was performed at 19+1 week to stabilize the uterine environment. The result was a successful extension of the pregnancy to 39+5 weeks, culminating in the birth of a healthy newborn. The conclusion indicates that this combined treatment strategy significantly extended the gestational period and reduced the risk of preterm birth.

This study highlights the potential advantages of combining fetal reduction with emergency cervical cerclage in the management of complex twin pregnancies. This strategy not only effectively prolongs gestation but also reduces the incidence of preterm-related complications, providing new clinical evidence for improving the survival quality of the remaining fetus. As illustrated in Figure 4, the combined approach of fetal reduction and emergency cervical cerclage has demonstrated significant success in extending the gestational period in twin pregnancies. In this case, the management of PROM at 16+6 weeks, followed by fetal reduction at 17+6 weeks and emergency cervical cerclage at 19+1 week, successfully extended the pregnancy to 39+5 weeks, resulting in the birth of a healthy newborn. This figure summarizes the study’s background, procedures, and conclusions, demonstrating the potential clinical application of this treatment strategy (Figure 4). By combining fetal reduction and cervical cerclage, this approach not only addresses the immediate threat of preterm birth but also provides obstetricians with a new method for managing high-risk multiple pregnancies. This strategy can potentially improve perinatal outcomes, especially in cases where existing standard treatments are less effective (Cheung et al., 2020; McDonnell and Martin, 2022). However, the limitation of this study lies in its nature as a single case report, lacking support from a large-scale sample dataset. This may restrict the generalizability of the findings, making them potentially inapplicable to broader populations. Therefore, the generalizability and reproducibility of these findings in other patient populations require further validation. Successful implementation of this strategy necessitates consideration of various factors, including the patient’s specific clinical presentation, the expertise of the medical team, and the resources available at the healthcare facility. Additionally, the ethical and psychological issues associated with fetal reduction must be carefully considered. The patient’s psychological state, social support, and understanding of the procedure can all influence the treatment’s outcome (Bardin et al., 2022; van de Mheen et al., 2015).

Future research should focus on large-scale, multicenter clinical trials to validate the efficacy and safety of the combined use of fetal reduction and cervical cerclage. Additionally, the exploration of novel tocolytic drugs and more advanced surgical techniques could further optimize the management strategies for multiple pregnancies. As a crucial treatment component, psychological support warrants further investigation to enhance patient compliance and quality of life. Establishing standardized management guidelines will help improve clinical practice consistency and increase the treatment success rate. Furthermore, conducting follow-up studies on long-term maternal and neonatal outcomes is essential for assessing the comprehensive impact of this strategy (Greenberg et al., 2020; Kristensen et al., 2023).

In summary, DID may be an effective treatment option when obstetricians face the delivery of one fetus in a twin pregnancy, as it can improve the survival rate of the remaining fetus and reduce morbidity. However, parents should be informed of this procedure’s potential risks and benefits. It is recommended that standardized management protocols for DID be established as soon as possible. In this report, we present a rare case where proactive fetal reduction combined with emergency cervical cerclage successfully allowed the remaining fetus in a twin pregnancy, which did not experience PROM, to be delivered after a 160-day latency period. This case may represent the longest successful DID case recorded to date.

## Conclusion

This study reports a case of dichorionic diamniotic twin pregnancy in which one fetus experienced PROM at 16+6 weeks. By employing fetal reduction and emergency cervical cerclage, the gestation of the second fetus was successfully extended to 39+5 weeks, resulting in a total extension of 160 days Figure 4 summarizes the treatment strategy used in this study, demonstrating that the combination of fetal reduction and emergency cervical cerclage significantly prolonged the pregnancy and reduced the risk of preterm birth. These findings suggest that, in cases of PROM in dichorionic diamniotic twin pregnancies, the combined approach of proactive fetal reduction and emergency cervical cerclage may be an effective clinical strategy to extend gestation and mitigate the risk of preterm birth.

However, as this study is based on a single case report, the generalizability and reproducibility of the results require validation through larger-scale studies. Future research should focus on collecting more similar cases and conducting comparative studies to verify the effectiveness and safety of this strategy. Additionally, the exploration of other potential tocolytic strategies and advanced surgical techniques is necessary to further optimize the management of multiple pregnancies. Furthermore, research should address the psychological support and ethical considerations following fetal reduction to ensure comprehensive and compassionate medical care.

In conclusion, this case report highlights the potential advantages of combining fetal reduction with emergency cervical cerclage in managing complex twin pregnancies. While the study is based on a single case, larger-scale research is needed to confirm its generalizability and safety. Nonetheless, it provides valuable clinical insights and data to support the future management of multiple pregnancies.

## Data Availability

The original contributions presented in the study are included in the article/supplementary material, further inquiries can be directed to the corresponding author.

## Abbreviations

ART, Assisted Reproductive Technologies; ACOG, American College of Obstetricians and Gynecologists; CRP, C-Reactive Protein; DID, Delayed Interval Delivery; LOA, left Occipitoanterior; NEC, Necrotizing Enterocolitis; NST, Non-Stress Test; PROM, Premature Rupture of Membranes; RDS, Respiratory Distress Syndrome.

## References

[B1] Abrams R. H. (1957). Double pregnancy; report of a case with thirty-five days between deliveries. Obstetrics Gynecol. 9 (4), 435–438. 13419262

[B2] ACOG (2014). ACOG Practice Bulletin No. 144: multifetal gestations: twin, triplet, and higher-order multifetal pregnancies. Obstetrics Gynecol. 123 (5), 1118–1132. 10.1097/01.AOG.0000446856.51061.3e 24785876

[B3] American College of Obstetricians and Gynecologists’ Committee on Practice Bulletins—Obstetrics (2016). Practice bulletin No. 171: management of preterm labor. Obstetrics Gynecol. 128 (4), e155–e164. 10.1097/AOG.0000000000001711 27661654

[B4] Arabin B. van Eyck J. (2009). Delayed-interval delivery in twin and triplet pregnancies: 17 years of experience in 1 perinatal center. Am. J. obstetrics Gynecol. 200 (2), 154.e1–e8. 10.1016/j.ajog.2008.08.046 19110229

[B5] Bardin R. Gupta M. Greenberg G. Nandrajog A. Tenenbaum-Gavish K. Gupta N. (2022). Fetal reduction from twin to singleton gestation: a meta-analysis. Int. J. Gynaecol. obstetrics official organ Int. Fed. Gynaecol. Obstetrics 158 (2), 260–269. 10.1002/ijgo.14016 34758109

[B6] Bell E. F. Hintz S. R. Hansen N. I. Bann C. M. Wyckoff M. H. DeMauro S. B. Eunice Kennedy Shriver National Institute of Child Health and Human Development Neonatal Research Network (2022). Mortality, in-hospital morbidity, care practices, and 2-year outcomes for extremely preterm infants in the US, 2013-2018. JAMA 327 (3), 248–263. 10.1001/jama.2021.23580 35040888 PMC8767441

[B7] Benito Vielba M. De Bonrostro Torralba C. Pallares Arnal V. Herrero Serrano R. Tejero Cabrejas E. L. Campillos Maza J. M. (2019). Delayed-interval delivery in twin pregnancies: report of three cases and literature review. Int. Soc. Perinat. Obstetricians 32 (2), 351–355. 10.1080/14767058.2017.1378336 28889767

[B8] Chen M. Su F. Wang J. Zhou L. Liu Q. Chai X. (2021). Temporal persistence of residual fetal cell-free DNA from a deceased cotwin after selective fetal reduction in dichorionic diamniotic twin pregnancies. Prenat. Diagn. 41 (12), 1602–1610. 10.1002/pd.5898 33555061

[B9] Cheong M. A. Tay S. K. (2014). Application of legal principles and medical ethics: multifetal pregnancy and fetal reduction. Singap. Med. J. 55 (6), 298–301. 10.11622/smedj.2014077 PMC4294055 25017403

[B10] Cheung K. W. Seto M. T. Y. Wang W. Lai C. W. S. Kilby M. D. Ng E. H. Y. (2020). Effect of delayed interval delivery of remaining fetus(es) in multiple pregnancies on survival: a systematic review and meta-analysis. Am. J. obstetrics Gynecol. 222 (4), 306–319. 10.1016/j.ajog.2019.07.046 31394069

[B11] Cristinelli S. Fresson J. André M. Monnier-Barbarino P. (2005). Management of delayed-interval delivery in multiple gestations. Fetal diagnosis Ther. 20 (4), 285–290. 10.1159/000085087 15980642

[B12] Cui H. Li H. Yin Z. (2024). Emergency cervical cerclage in delayed-interval delivery of twin pregnancies: a scoping review. BMC pregnancy childbirth 24 (1), 323. 10.1186/s12884-024-06515-x 38671355 PMC11046782

[B13] da Silva Lopes K. Takemoto Y. Ota E. Tanigaki S. Mori R. (2017). Bed rest with and without hospitalisation in multiple pregnancy for improving perinatal outcomes. Cochrane database Syst. Rev. 3 (3), CD012031. 10.1002/14651858.CD012031.pub2 28262917 PMC6464520

[B14] de Frias C. A. S. Queirós A. S. P. A. F. Simões H. T. F. (2020). Delayed-interval delivery in dichorionic twin pregnancies: a case report of 154 latency days. Rev. Bras. Ginecol. Obstet. Rev. Fed. Bras. das Soc. Ginecol. Obstet. 42 (1), 61–64. 10.1055/s-0040-1701468 PMC10316835 32107767

[B15] Feys S. Jacquemyn Y. (2016). Delayed-interval delivery can save the second twin: evidence from a systematic review. Facts, views and Vis. ObGyn 8 (4), 223–231. PMC5303700 28210482

[B16] Gestations M. Twin T. (2021). Higher-order multifetal pregnancies: ACOG practice bulletin, number 231. Obstetrics Gynecol. 137 (6), e145–e162. 10.1097/AOG.0000000000004397 34011891

[B17] Greenberg G. Bardin R. Danieli-Gruber S. Tenenbaum-Gavish K. Shmueli A. Krispin E. (2020). Pregnancy outcome following fetal reduction from dichorionic twins to singleton gestation. BMC pregnancy childbirth 20 (1), 389. 10.1186/s12884-020-03076-7 32620088 PMC7333296

[B18] Haas D. M. Caldwell D. M. Kirkpatrick P. McIntosh J. J. Welton N. J. (2012). Tocolytic therapy for preterm delivery: systematic review and network meta-analysis. BMJ Clin. Res. ed. 345, e6226. 10.1136/bmj.e6226 PMC4688428 23048010

[B19] Hessami K. Evans M. I. Nassr A. A. Espinoza J. Donepudi R. V. Cortes M. S. (2022). Fetal reduction of triplet pregnancies to twins vs singletons: a meta-analysis of survival and pregnancy outcome. Am. J. obstetrics Gynecol. 227 (3), 430–439.e5. 10.1016/j.ajog.2022.03.050 35351408

[B20] Kaneko M. Kawagoe Y. Oonishi J. Yamada N. Sameshima H. Ikenoue T. (2012). Case report and review of delayed-interval delivery for dichorionic, diamniotic twins with normal development. J. obstetrics Gynaecol. Res. 38 (4), 741–744. 10.1111/j.1447-0756.2011.01761.x 22380468

[B21] Khalil A. Liu B. (2021). Controversies in the management of twin pregnancy. Ultrasound obstetrics and Gynecol. official J. Int. Soc. Ultrasound Obstetrics Gynecol. 57 (6), 888–902. 10.1002/uog.22181 32799348

[B22] Khalil A. Prasad S. (2022). Screening and prevention of preterm birth in twin pregnancies. Best Pract. and Res. Clin. obstetrics and Gynaecol. 84, 179–193. 10.1016/j.bpobgyn.2022.08.008 36180317

[B23] Kolben T. Fischer D. Ruehl I. Franz M. Hester A. Kolben T. M. (2019). Delayed interval delivery in multiple gestations: the Munich experience. Archives Gynecol. obstetrics 299 (2), 339–344. 10.1007/s00404-018-4959-2 30386991

[B24] Kristensen S. E. Ekelund C. K. Sandager P. Jørgensen F. S. Hoseth E. Sperling L. (2023). Risks and pregnancy outcome after fetal reduction in dichorionic twin pregnancies: a Danish national retrospective cohort study. Am. J. obstetrics Gynecol. 228 (5), 590.e1–590.e12. 10.1016/j.ajog.2022.10.028 36441092

[B25] Louchet M. Dussaux C. Luton D. Goffinet F. Bounan S. Mandelbrot L. (2020). Delayed-interval delivery of twins in 13 pregnancies. J. Gynecol. obstetrics Hum. reproduction 49 (2), 101660. 10.1016/j.jogoh.2019.101660 31809959

[B26] McDonnell B. P. Martin A. (2022). Delayed interval delivery of preterm multiples: experience from a large specialized twin center. Int. Soc. Perinat. Obstetricians 35 (12), 2227–2233. 10.1080/14767058.2020.1782375 32586161

[B27] Ohuma E. O. Moller A. B. Bradley E. Chakwera S. Hussain-Alkhateeb L. Lewin A. (2023). National, regional, and global estimates of preterm birth in 2020, with trends from 2010: a systematic analysis. Lancet London, Engl. 402 (10409), 1261–1271. 10.1016/S0140-6736(23)00878-4 37805217

[B28] Porreco R. P. Sabin E. D. Heyborne K. D. Lindsay L. G. (1998). Delayed-interval delivery in multifetal pregnancy. Am. J. obstetrics Gynecol. 178 (1 Pt 1), 20–23. 10.1016/s0002-9378(98)70620-9 9465797

[B29] Raposo M. I. Cardoso M. Ormonde M. Stokreef S. Correia L. Pereira A. (2017). Obstetric management of delayed-interval delivery. Case Rep. women's health 16, 11–13. 10.1016/j.crwh.2017.09.002 29594002 PMC5842964

[B30] Roman A. Ramirez A. Fox N. S. (2022). Prevention of preterm birth in twin pregnancies. Am. J. obstetrics and Gynecol. MFM 4 (2S), 100551. 10.1016/j.ajogmf.2021.100551 34896357

[B31] Sunderam S. Kissin D. M. Zhang Y. Jewett A. Boulet S. L. Warner L. (2022). Assisted reproductive technology surveillance - United States, 2018. Surveill. Summ. 71 (4), 1–19. 10.15585/mmwr.ss7104a1 PMC8865855 35176012

[B32] van de Mheen L. Everwijn S. M. Knapen M. F. Haak M. C. Engels M. A. Manten G. T. (2015). Pregnancy outcome after fetal reduction in women with a dichorionic twin pregnancy. Hum. Reprod. Oxf. Engl. 30 (8), 1807–1812. 10.1093/humrep/dev132 26093542

[B33] Vieira L. A. Warren L. Pan S. Ferrara L. Stone J. L. (2019). Comparing pregnancy outcomes and loss rates in elective twin pregnancy reduction with ongoing twin gestations in a large contemporary cohort. Am. J. obstetrics Gynecol. 221 (3), 253.e1–253. 10.1016/j.ajog.2019.04.001 30995460

[B34] Weitzner O. Barrett J. Murphy K. E. Kingdom J. Aviram A. Mei-Dan E. (2023). National and international guidelines on the management of twin pregnancies: a comparative review. Am. J. obstetrics Gynecol. 229 (6), 577–598. 10.1016/j.ajog.2023.05.022 37244456

[B35] Yang S. Y. Liu C. Hsieh P. L. (2022). Effects of team-based learning on students' teamwork, learning attitude, and health care competence for older people in the community to achieve SDG-3. Int. J. Environ. Res. public health 19 (11), 6632. 10.3390/ijerph19116632 35682217 PMC9180350

[B36] Yodoshi T. Tipton E. Rouse C. A. (2015). A case of delayed interval delivery with a successful hospital move. Case Rep. Pediatr. 2015, 802097. 10.1155/2015/802097 26413366 PMC4568053

[B37] Zhang J. Johnson C. D. Hoffman M. (2003). Cervical cerclage in delayed interval delivery in a multifetal pregnancy: a review of seven case series. Eur. J. obstetrics, Gynecol. reproductive Biol. 108 (2), 126–130. 10.1016/s0301-2115(02)00479-7 12781398

[B38] Zhang J. Wei Z. Zhang Z. (2022). Correlation of poly (adenosine diphosphate[ADP]-ribose) polymerase expression and prognosis in ovarian cancer: a systematic review and meta-analysis. J. Gynecol. Obstet. Hum. Reprod. 51 (4), 102344. 10.1016/j.jogoh.2022.102344 35218983

[B39] Zheng X. Q. Yan J. Y. Xu R. L. Wang X. C. Li L. Y. Lin Z. (2020). An analysis of the maternal and infant outcomes in the delayed interval delivery of twins. Taiwan. J. obstetrics and Gynecol. 59 (3), 361–365. 10.1016/j.tjog.2020.03.004 32416880

